# Growth and tolerance of healthy, term infants fed lower protein extensively hydrolyzed or amino acid-based formula: double-blind, randomized, controlled trial

**DOI:** 10.1186/s12887-021-02617-z

**Published:** 2021-07-21

**Authors:** Craig B. Adams, William H. Johnston, Harold Deulofeut, Joseph Leader, Robbie Rhodes, Michael Yeiser, Cheryl L. Harris, Jennifer L. Wampler, Rebecca J. Hill, Timothy Cooper

**Affiliations:** 1 Southeastern Pediatric Associates, 364 Honeysuckle Road, Dothan, AL 36305 USA; 2 Birmingham Pediatric Associates, 806 St Vincent’s Drive, Suite 615, Birmingham, AL 35205 USA; 3 Children’s Medical Association, 8430 W Broward Blvd, Plantation, FL 33324 USA; 4 Woburn Pediatric Associates Research Dept, 7 Alfred Street, Suite 220, Woburn, MA 01801 USA; 5 Central Arkansas Pediatric Clinic, 2301 Springhill Road, Suite 200, Benton, AR 72019 USA; 6 Owensboro Pediatrics, 2200 East Parrish Ave, Owensboro, KY 42303 USA; 7 Medical Affairs and Scientific Affairs, Reckitt/Mead Johnson Nutrition Institute, Evansville, IN 47721 USA

**Keywords:** Infant formula, Extensively hydrolyzed protein, Amino acid, *Lactobacillus rhamnosus* GG

## Abstract

**Background:**

Optimal protein level in hypoallergenic infant formulas is an area of ongoing investigation. The aim was to evaluate growth of healthy term infants who received extensively hydrolyzed (EH) or amino acid (AA)-based formulas with reduced protein.

**Methods:**

In this prospective, multi-center, double-blind, controlled, parallel group study, infants were randomized to receive a marketed EH casein infant formula at 2.8 g protein/100 kcal (Control) or one of two investigational formulas: EH casein formula at 2.4 g protein/100 kcal (EHF) or AA-based formula at 2.4 g total protein equivalents/100 kcal (AAF). Control and EHF each had 2 × 10^7^ CFU *Lactobacillus rhamnosus* GG/100 kcal. Anthropometrics were measured and recall of formula intake, tolerance, and stool characteristics was collected at 14, 30, 60, 90, 120 days of age. Primary outcome was weight growth rate (g/day) between 14 and 120 days of age (analyzed by ANOVA). Medically confirmed adverse events were recorded throughout the study.

**Results:**

No group differences in weight or length growth rate from 14 to 120 days were detected. With the exception of significant differences at several study time points for males, no group differences were detected in mean head circumference growth rates. However, mean achieved weight, length, and head circumference demonstrated normal growth throughout the study period. No group differences in achieved weight or length (males and females) and head circumference (females) were detected and means were within the WHO growth 25th and 75th percentiles from 14 to 120 days of age. With the exception of Day 90, there were no statistically significant group differences in achieved head circumference for males; means remained between the WHO 50th and 75th percentiles for growth at Days 14, 30, and 60 and continued along the 75th percentile through Day 120. No differences in study discontinuation due to formula were detected. The number of participants for whom at least one adverse event was reported was similar among groups.

**Conclusions:**

This study demonstrated hypoallergenic infant formulas at 2.4 g protein/100 kcal were safe, well-tolerated, and associated with appropriate growth in healthy term infants from 14 to 120 days of age.

**Trial registration:**

ClinicalTrials.gov, ClinicalTrials.gov Identifier: NCT01354366. Registered 13 May 2011.

## Background

Cow’s milk allergy (CMA) is one of the most common allergies in infancy, with a clinically diagnosed prevalence estimated up to 3% (as reviewed [1–5]). Hypoallergenic formulas, including extensively hydrolyzed (EH) protein formulas or amino acid-based (AA) formulas, are recommended for the dietary management of infants with CMA who cannot be breastfed. Typically, the majority of infants with CMA are managed effectively on an EH formula, however, approximately 10% of infants who exhibit severe CMA and/or multiple food allergies will require dietary management utilizing an AA-based formula [6].

Increasing evidence supports lowering total protein content in both hypoallergenic formulas and routine infant formulas to be more in line with the amount of protein found in human milk. Human milk is dynamic in composition and declines in protein content as lactation progresses [7] whereas infant formula composition is static for each age stage and formulated with higher protein concentrations than human milk to meet essential AA requirements [8, 9]. Targeting a lower protein concentration in infant formulas, more similar to that of human milk in order to support growth in line with breastfed trajectories, has increasing support [9–11]. As a result, some regulatory recommendations have been updated for infant formula protein composition by lowering the maximum target value. For example, the European Union has recently lowered its maximum recommended value for protein hydrolysates in infant formula from 3 g/100 kcal [12] to 2.8 g/100 kcal [13].

Though management of CMA is the primary goal of EH or AA formula usage, a reduction of protein in infant formula requires consideration with respect to growth and tolerance. The overall importance of adequate protein for infant growth and development is well understood. Both an EH and an AA-based formula that had protein at 2.8 g/100 kcal have been demonstrated to adequately support typical growth and safety [14]. In the current study we aimed to examine the growth and tolerance of healthy, term infants fed an EH or AA formula that have reduced protein/protein equivalent at 2.4 g/100 kcal. Rate of weight gain (g/day) from 14 to 120 days of age was evaluated as the primary variable to establish that protein at this concentration is well-accepted, tolerated and provides adequate growth.

## Methods

### Study design

Good Manufacturing Practice guidelines are provided by the US Food and Drug Administration to insure that an infant formula meets the quality factor of normal physical growth [15, 16]. Consequently, the current multicenter, double-blind, randomized, controlled, parallel-group, prospective trial is similar in design to previously reported studies [17, 18] in order to report consistent growth outcomes across different study cohorts. The research protocol and informed consent forms observing the Declaration of Helsinki (including October 1996 amendment) were approved by: the University of Louisville Institutional Review Board (IRB; Louisville, KY); the University of Nebraska Medical Center IRB (Omaha, NE); Western IRB (Olympia, WA); and Schulman IRB (now known as Advarra, Columbia, MD). The study complied with good clinical practices. Mothers who had decided to exclusively provide infant formula were screened for study eligibility. Parents or legally authorized representatives provided written informed consent prior to enrollment.

Healthy 12- to 16-day old infants were recruited at 27 clinical sites in the United States. Eligible infants were singleton births at 37–42 weeks’ gestational age with birth weight ≥ 2500 g and solely receiving infant formula at least 24 h prior to randomization. Exclusion criteria included history of underlying disease or congenital malformation likely to interfere with normal growth and development or participant evaluation; feeding difficulties or history of formula intolerance; weight at randomization < 98% of birth weight; large for gestational age from mother who was diabetic at childbirth; and immunodeficiency.

A computer-generated, randomization schedule stratified by sex was created by the study sponsor and provided in sealed consecutively numbered envelopes for each study site. At each study site the next sequential envelope was opened from the appropriate set to assign study formula. Two unique codes (known only to the sponsor) were used to designate each study formula. Study formulas were dispensed to parents at each study visit prior to completion or withdrawal. Product labels and the sealed envelopes did not allow direct unblinding by the study site. Study monitoring personnel were also blinded to study product identification. In the event of a medical emergency (in which knowledge of the study formula was critical to the participant’s management) blinding for a participant could be broken by study sponsor personnel. In this study, it was not necessary to break the study code prematurely.

Participants were enrolled between July 2011 and August 2012 and were randomly assigned to receive one of three study formulas (Mead Johnson Nutrition, Evansville, IN) from 14 to 120 days of age: 1) an EH casein infant formula at 2.8 g protein/100 kcal (Control; marketed Nutramigen™ with Enflora™ LGG®); 2) an investigational EH casein formula at 2.4 g protein/100 kcal (EHF); or 3) an investigational AA-based formula at 2.4 g total protein equivalents/100 kcal (AAF). Control and EHF each had 2 × 10^7^ CFU *Lactobacillus rhamnosus* GG (LGG)/100 kcal (Table 1).

**Table 1:** Nutrient composition per 100 kcal

Nutrient	Study Formula, target values		
	Control^a^	EHF^a,b^	AAF
Total Protein, g^c^	2.8	2.4	–
Protein Equivalent, g^d^	–	–	2.4
Total Fat, g^e^	5.3	5.3	5.3
Linoleic acid, mg	860	860	860
α-Linolenic acid, mg	80	80	80
ARA, mg	34	34	34
DHA, mg	17	17	17
Total Carbohydrate, g^f^	10.3	10.7	10.7
Vitamin A, IU	300	300	300
Vitamin D, IU	50	50	50
Vitamin E, IU	2	2	2
Vitamin K, mcg	9	9	8
Thiamin, mcg	80	80	80
Riboflavin, mcg	90	90	90
Vitamin B6, mcg	60	60	60
Vitamin B12, mcg	0.3	0.3	0.3
Niacin, mcg	1000	1000	1000
Folic Acid, mcg	16	16	16
Pantothenic Acid, mcg	500	500	500
Biotin, mcg	3	3	3
Vitamin C, mg	12	12	12
Choline, mg	24	24	24
Inositol, mg	17	17	17
Calcium, mg	94	94	94
Phosphorus, mg	52	52	52
Magnesium, mg	8	8	11
Iron, mg	1.8	1.8	1.8
Zinc, mg	1	1	1
Manganese, mcg	25	25	60
Copper, mcg	75	75	75
Iodine, mcg	15	15	15
Selenium, mcg	2.8	2.8	2.8
Sodium, mg	47	41	43
Potassium, mg	110	104	110
Chloride, mg	86	81	79

^a^ *Lactobacillus rhamnosus* GG

^b^ Sources of protein: casein hydrolysate, L-cystine, L-tyrosine, L-tryptophan

^c^ Exempt infant formula [16]

^d^ 100% Free amino acids

^e^ Sources of fat: Blend of palm olein, soy, coconut, and high oleic sunflower oils; single cell oils as a source of ARA and DHA

^f^ Sources of carbohydrate for Control and EHF: corn syrup solids, modified corn starch and for AAF: corn syrup solids, modified tapioca starch

### Study objectives and outcomes

Evaluation of growth and tolerance in healthy, term infants was the study objective. Body weight, length, and head circumference (anthropometric measures) were recorded at study visits corresponding to 14 (12–16 days; enrollment), 30 (±3), 60 (±3), 90 (±3), 120 (±4) days of age.

At all study visits parents completed a 24-h recall of tolerance (fussiness and gassiness) and stool characteristics (frequency and consistency); study formula intake (fluid oz./day) was reported beginning at the 30 days of age visit. We have previously characterized tolerance using the same reporting scales [17, 18, 19, 20, 21]Although parental recall may vary among individuals, participant randomization ensures balance between study groups. Responses were scaled for amount of gas (none = 0, slight amount = 1, moderate amount = 2, excessive amount = 3); fussiness (not fussy = 0, slightly fussy = 1, moderately fussy = 2, very fussy = 3, extremely fussy = 4); and stool consistency (hard = 1, formed = 2, soft = 3, unformed or seedy, watery = 4). Each participant’s parent or caregiver was provided with pictures to guide in stool consistency rating. Adverse events were coded according to specific event and the body system involved.

### Statistical methods

Weight growth rate from 14 to 120 days of age was the primary outcome. Detection of a clinically relevant difference of 3 g/day in weight gain from 14 to 120 days of age (80% power) was used to determine the sample size. Enrollment of approximately 91 males and 66 females was needed in each group with expected completion of 59 male and 43 female participants per study group (assuming a standard deviation of 6.5 g/day for male and 5.5 g/day for female participants). Linear regression of weight on age was calculated for each participant and analysis of variance (ANOVA) was used to assess growth rates from 14 to 30, 60, 90, or 120 days of age. A one-tailed test, as outlined in guidance provided by the American Academy of Pediatrics (AAP) Task Force on Clinical Testing of Infant Formulas [22] was used to compare mean weight growth rates by sex for the investigational formula groups with the control.

Secondary outcomes included other anthropometric and tolerance measures and medically confirmed adverse events. Participant characteristics (race, ethnicity, sex, family history of allergy) and adverse events were analyzed by Fisher’s exact test. Achieved weight, length, and head circumference; length and head circumference growth rates; formula intake; and stool frequency were analyzed by ANOVA with two exceptions. For females, because a difference in head circumference was detected at enrollment, analysis of covariance (ANCOVA) with covariate head circumference at enrollment was used to analyze head circumference growth rates and achieved head circumference. Stool consistency, fussiness, and gas were analyzed using the Cochran-Mantel-Haenszel (CMH) row mean score test. With the exception of *p*-values reported from the analysis of weight gain based on one-tailed tests, all other *p*-values reported were based on two-tailed tests. When the overall comparison of the three groups was significant, unadjusted pairwise group comparisons were performed. All testing was conducted at α = 0.05. All analyses were conducted using SAS version 9.2 (Cary, NC).

## Results

### Participants

**Fig. 1:**
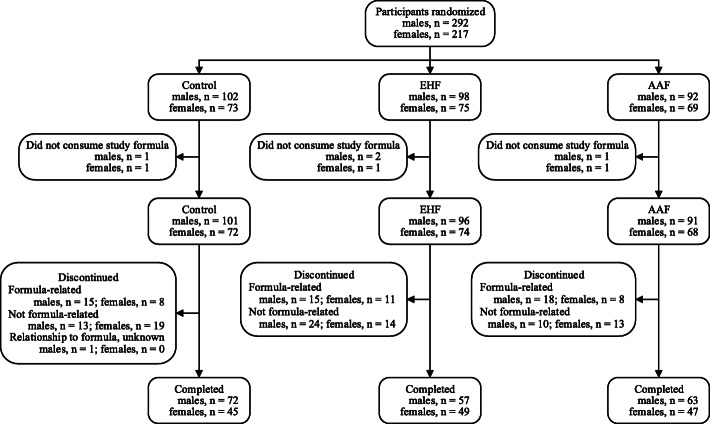
Flow of study participants

A total of 509 participants were enrolled and randomized (Control: 175; EHF: 173; AAF: 161). Participants who were randomized but consumed no study formula (Control: 2; EHF: 3; AAF: 2) were not included in subsequent analyses (Fig. 1). Anthropometric measures at birth, family history of allergy, and sex, race, and ethnic distribution were similar among groups (data not shown). With the exception of significantly lower mean (±SE) head circumference for female infants in the Control versus the EHF group (35.5 ± 0.1 vs 36.0 ± 0.1 cm; *P* = 0.004), no differences in body weight or length, or head circumference by sex were observed among groups at study enrollment (Table 2). No statistically significant group differences were detected for overall study discontinuation (Control: 56, 32%; EHF: 64, 38%; AAF: 49, 31%) or discontinuation related to study formula (Control: 23, 13%; EHF: 26, 15%; AAF: 26, 16%). In the total study population, 52 (10%) participants (Control: 17; EHF: 18; AAF: 17) discontinued due to formula intolerance as determined by the study investigator; fussiness (Control: 10; EHF: 10; AAF: 7) was the most common indicator. Parental decision was the most common reason for discontinuation unrelated to study formula (67 participants, 13%). A total of 333 infants completed the study (Control: 117; EHF: 106; AAF: 110).

**Table 2:** Infant characteristics at study entry

	Study Group			Overall
	Control	EHF	AAF	*P*
Total number of participants	173	170	159	0.935
males/females, n	101/72	96/74	91/68	
*males*^a^				
Weight (g)	3730.9 ± 44.3	3689.8 ± 45.4	3684.7 ± 46.6	0.729
Length (cm)	52.7 ± 0.2	52.5 ± 0.2	52.4 ± 0.2	0.638
Head circumference (cm)	36.4 ± 0.1	36.1 ± 0.1	36.2 ± 0.1	0.304
*females*^a^				
Weight (g)	3482.7 ± 45.8	3626.7 ± 45.2	3554.1 ± 47.1	0.084
Length (cm)	51.5 ± 0.2	52.0 ± 0.2	51.5 ± 0.2	0.176
Head circumference (cm)	35.5 ± 0.1†	36.0 ± 0.1	35.6 ± 0.1	0.013†

^a^ Mean ± standard error (SE)

†Significantly different, Control vs EHF (*P* = 0.004)

### Growth

**Fig. 2:**
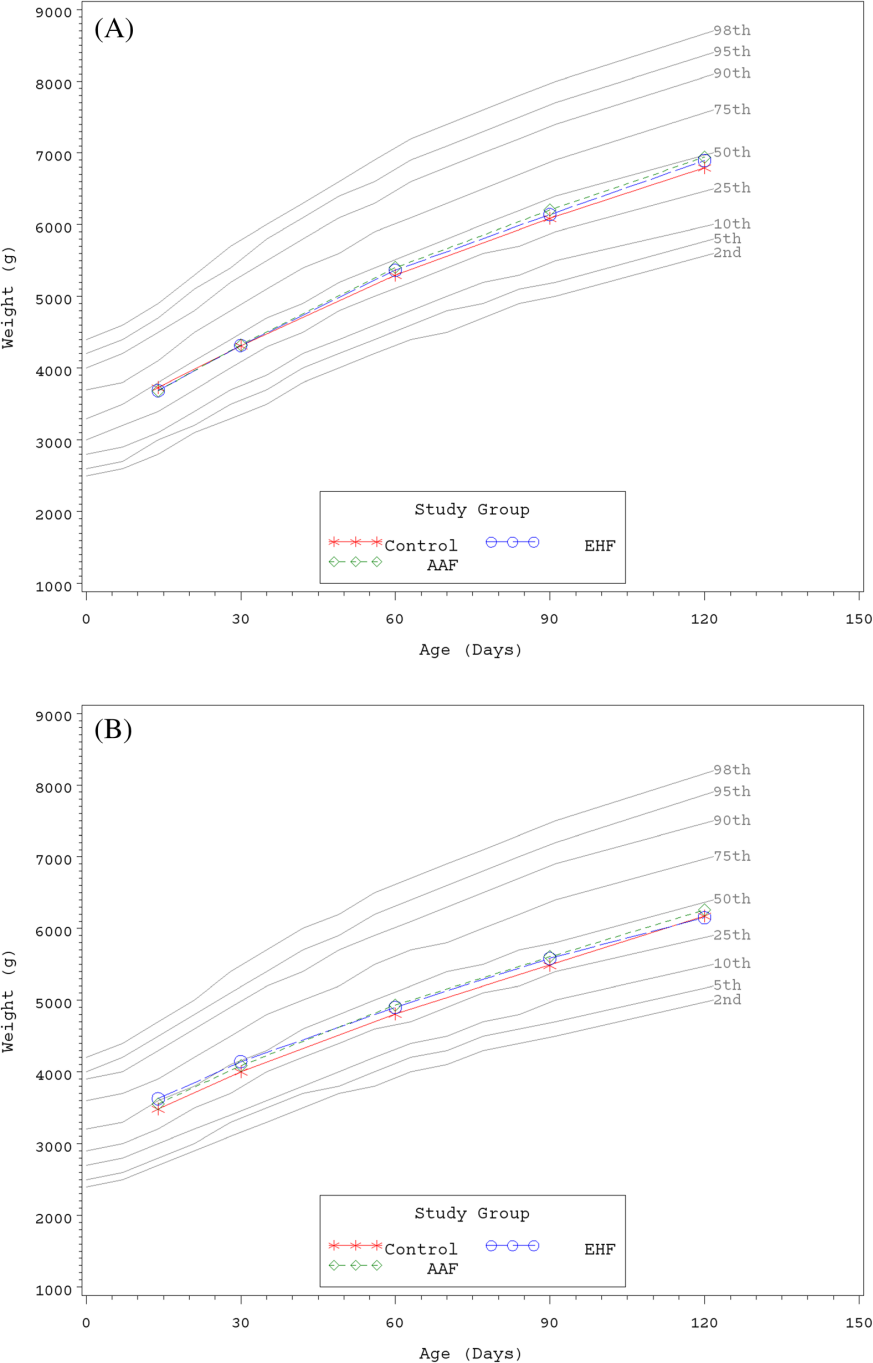
**a** Mean achieved weight for male participants with World Health Organization (WHO) percentiles (2nd to 98th) from 14 to 120 days of age. Control, stars; EHF, circles; AAF, diamonds. **b** Mean achieved weight for female participants with World Health Organization (WHO) percentiles (2nd to 98th) from 14 to 120 days of age. Control, stars; EHF, circles; AAF, diamonds

**Fig. 3:**
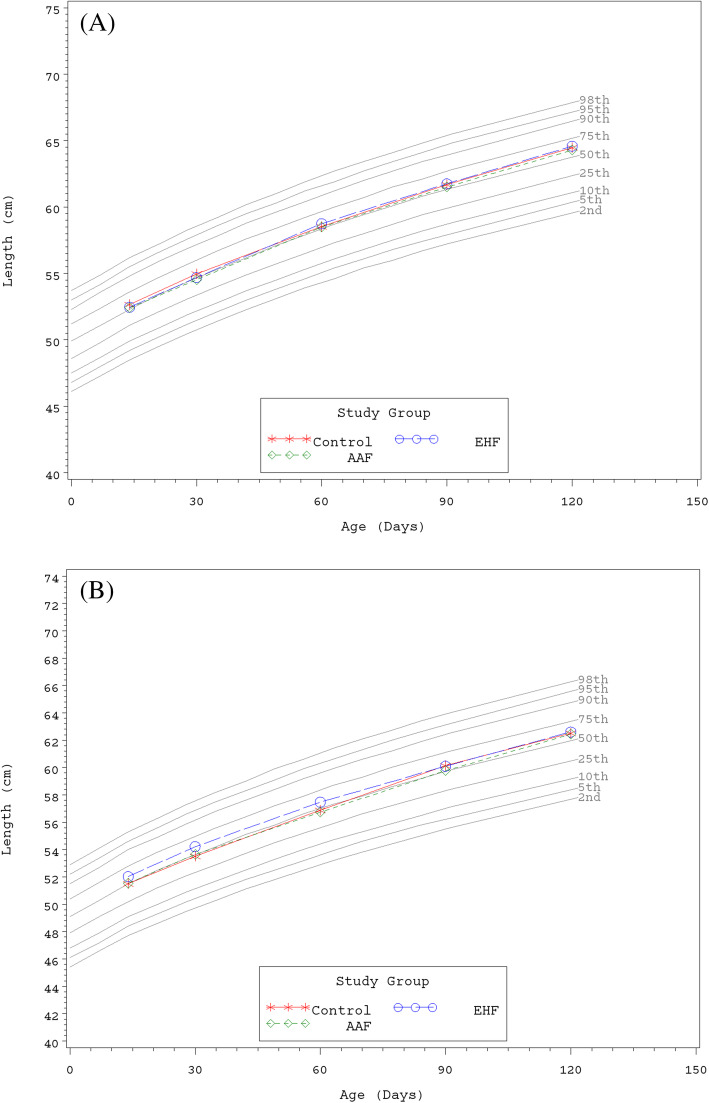
**a** Mean achieved length for male participants with World Health Organization (WHO) percentiles (2nd to 98th) from 14 to 120 days of age. Control, stars; EHF, circles; AAF, diamonds. **b** Mean achieved length for female participants with World Health Organization (WHO) percentiles (2nd to 98th) from 14 to 120 days of age. Control, stars; EHF, circles; AAF, diamonds

**Fig. 4:**
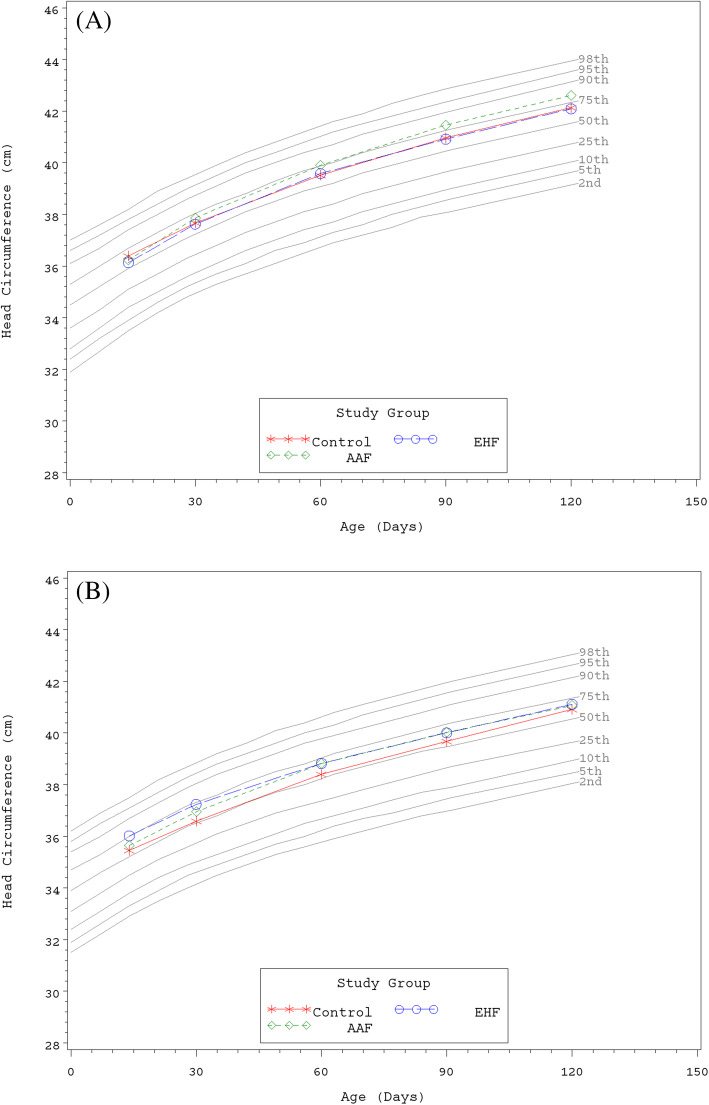
**a** Mean achieved head circumference for male participants with World Health Organization (WHO) percentiles (2nd to 98th) from 14 to 120 days of age. Control, stars; EHF, circles; AAF, diamonds. **b** Mean achieved head circumference for female participants with World Health Organization (WHO) percentiles (2nd to 98th) from 14 to 120 days of age. Control, stars; EHF, circles; AAF, diamonds

Growth rates were analyzed from 14 to 120 days of age. No statistically significant group differences in the primary outcome, weight growth rate from Day 14 to 120, were detected by sex (Table 3). No statistically significant group differences in weight or length growth rates were detected by sex for any age range. In addition, no significant group differences were observed for mean achieved weight or length at any measured time point. For all groups at all measured time points, mean achieved weights plotted within the 25th and 50th percentiles for male (Fig. 2a) and female (Fig. 2b) participants and mean achieved lengths plotted within the 50th and 75th percentiles for male (Fig. 3a) and female (Fig. 3b) participants using the WHO growth charts [23, 24]. For head circumference growth rate, significant group differences were detected for males including: higher growth rate in the EHF or AAF versus the Control group from Day 14 to 30; higher for AAF versus Control from Day 14 to 60; higher for AAF versus Control or EHF from Day 14 to 90; and higher for AAF versus the Control or EHF from Day 14 to 120 (Table 3). In addition for males, mean (±SE) achieved head circumference (cm) was significantly higher at Day 90 for AAF (41.5 ± 0.2) versus Control (41.0 ± 0.2; *P* = 0.033) or EHF (40.9 ± 0.2; *P* = 0.029); however, the means for all groups plotted within the 50th and 75th percentiles of WHO growth charts at Days 14, 30, and 60 and continued along the 75th percentile through Day 120 (Fig. 4a). For females, no significant differences in adjusted head circumference growth rates or achieved head circumference were detected. Mean achieved head circumferences for females in all groups plotted within the 50th and 75th percentiles of the WHO growth charts at all measured study time points (Fig. 4b).

**Table 3:** Weight, length, and head circumference growth rates from 14 days to 30, 60, 90, and 120 days of age

			Growth rate^a^	
Day	Group (n)	Weight (g/day)	Length (cm/day)	Head circumference (cm/day)
male				
30	Control (90)	35.3 ± 1.3	0.14 ± 0.008	0.076 ± 0.004*†
	EHF (80)	37.8 ± 1.4	0.13 ± 0.009	0.091 ± 0.004
	AAF (82)	39.7 ± 1.4	0.13 ± 0.009	0.098 ± 0.004
60	Control (78)	33.2 ± 1.0	0.12 ± 0.004	0.067 ± 0.002†
	EHF (67)	34.7 ± 1.1	0.13 ± 0.004	0.071 ± 0.002
	AAF (67)	36.4 ± 1.1	0.13 ± 0.004	0.076 ± 0.002
90	Control (74)	30.5 ± 0.8	0.11 ± 0.002	0.060 ± 0.001†
	EHF (60)	31.7 ± 0.9	0.12 ± 0.003	0.060 ± 0.002†
	AAF (64)	33.0 ± 0.9	0.12 ± 0.003	0.067 ± 0.001
120	Control (71)	28.6 ± 0.7	0.11 ± 0.002	0.054 ± 0.001†
	EHF (57)	29.5 ± 0.8	0.11 ± 0.002	0.054 ± 0.001†
	AAF (63)	30.5 ± 0.7	0.11 ± 0.002	0.059 ± 0.001
female				
30	Control (62)	31.6 ± 1.3	0.12 ± 0.009	0.075 ± 0.004
	EHF (58)	33.0 ± 1.4	0.14 ± 0.009	0.077 ± 0.005
	AAF (59)	32.9 ± 1.3	0.13 ± 0.009	0.081 ± 0.005
60	Control (55)	28.3 ± 1.0	0.11 ± 0.004	0.063 ± 0.002
	EHF (52)	28.1 ± 1.0	0.12 ± 0.004	0.063 ± 0.002
	AAF (50)	29.8 ± 1.0	0.11 ± 0.004	0.069 ± 0.002
90	Control (49)	26.0 ± 0.9	0.11 ± 0.003	0.055 ± 0.002
	EHF (50)	25.7 ± 0.9	0.11 ± 0.003	0.054 ± 0.002
	AAF (49)	26.8 ± 0.9	0.11 ± 0.003	0.058 ± 0.002
120	Control (45)	24.8 ± 0.8	0.10 ± 0.002	0.050 ± 0.001
	EHF (49)	24.2 ± 0.8	0.10 ± 0.002	0.049 ± 0.001
	AAF (46)	25.1 ± 0.8	0.10 ± 0.002	0.050 ± 0.001

^a^mean ± standard error (SE); adjusted mean ± SE for Head Circumference for females

*Significantly different vs EHF, *P* < 0.05

†Significantly different vs AAF, *P* < 0.05

### Tolerance

fvNo group differences in parent-reported gassiness and fussiness were detected at any time point assessed, with the exception of fussiness at Day 30 (Table 4). Mean stool frequency was lower in the AAF versus the Control or EHF groups at Day 30 (*P* < 0.001); lower in EHF or AAF versus Control at Day 60 (*P* ≤ 0.025); and lower in AAF versus Control or EHF at Day 90 (*P* ≤ 0.008) (Table 5). No significant group differences were detected in mean stool frequency at Day 120. Significant differences in stool consistency were detected in the AAF versus Control or EHF groups at Days 30, 60, 90, and 120 (*P* < 0.001). At each measured time point, more infants with “unformed or seedy” and fewer infants with a “hard” or “formed” stool consistency in the Control and EHF groups compared to the AAF group were the primary differences observed in stool consistency categories.

**Table 4:** Fussiness and gassiness at Days 14, 30, 60, 90, and 120

		Fussiness, n (%)						Gassiness, n (%)				
Day	Group	Not at all	Slightly	Moderately	Very	Extremely	*P*	None at all	Slight amount	Moderate amount	Excessive amount	*P*
14	Control	43 (25)	90 (52)	34 (20)	4 (2)	2 (1)	0.513	11 (6)	75 (43)	73 (42)	14 (8)	0.791
	EHF	52 (31)	85 (50)	26 (15)	6 (4)	1 (1)		12 (7)	72 (42)	73 (43)	13 (8)	
	AAF	40 (25)	81 (51)	34 (22)	1 (1)	2 (1)		14 (9)	64 (41)	72 (46)	8 (5)	
30	Control	16 (11)	67 (48)	43 (30)	14 (10)	1 (1)	0.029*†	9 (6)	59 (42)	64 (45)	9 (6)	0.611
	EHF	21 (17)	66 (54)	30 (25)	4 (3)	1 (1)		4 (3)	56 (46)	51 (41)	12 (10)	
	AAF	22 (18)	60 (49)	37 (30)	2 (2)	2 (2)		9 (7)	54 (44)	51 (41)	9 (7)	
60	Control	30 (24)	57 (45)	32 (25)	5 (4)	2 (2)	0.171	11 (9)	49 (39)	51 (40)	15 (12)	0.294
	EHF	36 (32)	52 (46)	21 (19)	3 (3)	1 (1)		8 (7)	55 (49)	44 (39)	6 (5)	
	AAF	28 (25)	53 (46)	26 (23)	5 (4)	2 (2)		7 (6)	56 (49)	46 (40)	5 (4)	
90	Control	46 (38)	55 (45)	19 (16)	0 (0)	1 (1)	0.874	19 (16)	60 (50)	37 (31)	5 (4)	0.850
	EHF	45 (43)	40 (38)	15 (14)	3 (3)	2 (2)		12 (11)	56 (53)	32 (30)	5 (5)	
	AAF	39 (35)	54 (48)	15 (13)	4 (4)	0 (0)		18 (16)	55 (49)	29 (26)	10 (9)	
120	Control	51 (44)	41 (35)	17 (15)	6 (5)	1 (1)	0.192	25 (22)	52 (45)	35 (30)	4 (3)	0.783
	EHF	49 (47)	41 (39)	10 (10)	4 (4)	1 (1)		21 (20)	57 (54)	24 (23)	3 (3)	
	AAF	35 (33)	46 (43)	20 (19)	5 (5)	0 (0)		25 (24)	47 (44)	32 (30)	2 (2)	

* Control vs EHF significantly different, *P* = 0.015

† Control vs AAF significantly different, *P* = 0.043

**Table 5:** Stool characteristics at Days 14, 30, 60, 90, and 120^a^

		Stool	Overall	Stool consistency, n (%)					Overall
Day	Group (*n*)	frequency^b^	*P*	hard	formed	soft	Unformed or seedy	watery	*P*
14	Control (173)	2.9 ± 0.2	0.352	3 ( 2)	5 (3 )	67 (40)	87 (52)	6 (4)	0.679
	EHF (170)	3.3 ± 0.2		2 (1)	8 (5)	74 (45)	76 (46)	6 (4)	
	AAF (156)	3.2 ± 0.2		2 (1)	4 (3)	69 (45)	75 (49)	4 (3)	
30	Control (140)	3.6 ± 0.2	< 0.001*†	3 (2)	5 (4)	74 (53)	53 (38)	5 (4)	< 0.001*†
	EHF (121)	3.4 ± 0.2		1 (1)	5 (4)	67 (54)	46 (37)	4 (3)	
	AAF (123)	2.5 ± 0.2		21 (18)	43 (36)	48 (40)	7 (6)	0 (0)	
60	Control (125)	3.0 ± 0.1	< 0.001*‡	1 (1)	3 (2)	67 (54)	47 (38)	6 (5)	< 0.001*†
	EHF (113)	2.5 ± 0.1		0 (0)	3 (3)	58 (53)	42 (38)	7 (6)	
	AAF (114)	2.2 ± 0.1		8 (7)	27 (25)	62 (56)	11 (10)	2 (2)	
90	Control (121)	2.5 ± 0.1	0.008*†	0 (0)	3 (3)	66 (58)	33 (29)	12 (11)	< 0.001*†
	EHF (104)	2.5 ± 0.2		0 (0)	6 (6)	55 (54)	38 (37)	3 (3)	
	AAF (112)	1.9 ± 0.1		4 (4)	19 (17)	74 (67)	12 (11)	1 (1)	
120	Control (116)	2.4 ± 0.1	0.356	2 (2)	6 (5)	64 (57)	34 (30)	7 (6)	< 0.001*†
	EHF (105)	2.3 ± 0.1		1 (1)	6 (6)	65 (63)	30 (29)	2 (2)	
	AAF (105)	2.1 ± 0.1		5 (5)	24 (23)	65 (63)	10 (10)	0 (0)	

^a^24-hour recall at study visits

^b^Mean ± standard error (SE)

* Control vs AAF significantly different, *P* < 0.05

† EHF vs AAF significantly different, *P* < 0.05

‡ Control vs EHF significantly different, *P* < 0.05

With the exception of lower formula intake (fl oz.; mean ± SE) at Day 60 for male infants in the Control (30.2 ± 1.0) versus the EHF (33.4 ± 1.1) or AAF (34.2 ± 1.1) groups (*P* ≤ 0.036), there were no significant group differences by sex detected at Days 30, 60, 90, or 120 (Table 6). In addition, mean reported intakes increased from Day 30 to 120 for all groups indicating normal intake for this time period. No group difference was detected in the number of participants for whom at least one medically confirmed adverse event was reported (Control: 136, 79%; EHF: 127, 75%; AAF: 127, 80%; *P* = 0.476). The incidence of adverse events categorized within Body as a Whole; Cardiovascular; Eyes, Ears, Nose, and Throat; Musculoskeletal; Respiratory; or Urogenital systems had no statistically significant group differences for specific events. In the Gastrointestinal (GI) System, there were no significant group differences in the most commonly reported specific adverse event, gastroesophageal reflux (Control: 42, 24%; EHF: 35, 21%; AAF: 32, 20%). The incidence of constipation was lower in the Control (14, 8%) or EHF (10, 6%) versus the AAF group (43, 27%; *P* < 0.001); the incidence of diarrhea was lower in the AAF (2, 1%) versus the Control (13, 8%) or EHF (10, 6%; *P* ≤ 0.036) groups. Within the Metabolic and Nutrition System, an overall significant difference was detected for poor weight gain (Control: 4, 2%; EHF: 0; AAF: 0; *P* = 0.036) but no specific pairwise group differences were detected. Within the Skin System, the incidence of diaper rash was significantly higher in the Control (26, 15%) versus the AAF (9, 6%; *P* = 0.007); no significant differences were detected compared to the EHF (16, 9%) group. Any medically confirmed adverse event was considered serious if it met one or more of the following criteria: resulted in death, was life-threatening, required inpatient hospitalization or prolongation of existing hospitalization, resulted in persistent or significant disability/incapacity, or was a congenital anomaly/birth defect. A total of 16 participants experienced serious adverse events (Control: 5, 3%; EHF: 5, 3%; AAF: 6, 4%). All serious adverse events were individually evaluated by study site physicians and each was determined unrelated to study formula.

**Table 6:** Mean study formula intake (fluid oz./day) at Days 30, 60, 90, and 120

Day	Group	males, n	Mean ± SE	Overall	females, n	Mean ± SE	Overall
				*P*			*P*
30	Control	84	27.1 ± 0.9	0.390	57	26.1 ± 0.7	0.976
	EHF	70	27.7 ± 1.0		53	26.3 ± 0.7	
	AAF	70	28.9 ± 1.0		53	26.2 ± 0.7	
60	Control	76	30.2 ± 1.0	0.017*†	50	29.5 ± 1.0	0.556
	EHF	62	33.4 ± 1.1		51	29.6 ± 1.0	
	AAF	66	34.2 ± 1.1		49	30.9 ± 1.0	
90	Control	73	34.1 ± 1.0	0.247	48	30.6 ± 1.2	0.324
	EHF	56	33.1 ± 1.2		49	32.2 ± 1.2	
	AAF	63	35.8 ± 1.1		49	33.2 ± 1.2	
120	Control	71	36.4 ± 1.2	0.921	45	33.3 ± 1.2	0.222
	EHF	57	37.0 ± 1.4		48	35.3 ± 1.1	
	AAF	61	37.0 ± 1.3		45	36.1 ± 1.2	

* Control vs EHF significantly different, *P* = 0.036

† Control vs AAF significantly different, *P* = 0.007

## Discussion

This study demonstrated that two investigational hypoallergenic formulas, an EH casein formula with added LGG (2.4 g protein/100 kcal) and an AA-based formula (2.4 g protein equivalent/100 kcal), were safe and well-tolerated when fed to healthy term infants from 14 to 120 days of age. Rate of weight gain (g/day), per guidance provided by the AAP Task Force on Clinical Testing of Infant Formulas [22], was used as the primary variable to assess the nutritional suitability of study formulas. No statistically significant group differences were observed for weight and length growth rates from 14 to 120 days of age. No group differences were detected in mean head circumference growth rates for females. Significant differences were observed for males: mean head circumference growth rate was higher for the AAF compared to Control at all time points and higher than the EHF group at Days 90 and 120. However, mean achieved weight, length, and head circumference plotted on WHO charts demonstrated normal growth throughout the study period. There were no group differences in achieved weight (males and females), length (males and females), and head circumference (females) and means were within the 25th and 75th percentiles of the WHO growth chart from 14 to 120 days of age. With the exception of Day 90, there were no statistically significant group differences in achieved head circumference for males; means remained between the 50th and 75th percentiles of growth by WHO standards at Days 14, 30, and 60 and continued along the 75th percentile through Day 120. In addition, all clinical outcomes related to head circumference growth were considered normal.

The randomized, double-blind, controlled design is a key strength of this study. One limitation could be that an AA-based formula at 2.8 g protein/100 kcal was not included as a study formula. However, we have previously reported that an AA-based formula (vs a control casein EH formula) and a casein EH formula with added LGG (versus a control casein EH formula with no added LGG) were safe and supported growth in healthy, term infants in studies of comparable design [14, 25]. The growth rates reported in these previous studies were similar to those demonstrated in the current study of reduced protein EHF and AAF formulations. In addition, no breastfed reference group was registered for comparison within this clinical trial.

It is well known that infants fed formula grow on a different trajectory than infants exclusively receiving breast milk [10, 11, 26]. Upper limit recommendations for total protein content for inclusion in infant formulas have been reduced in recent years with the premise of supporting infants receiving formula to grow more similarly to breastfed infants. In the current study, we have compared two reduced protein formulas to an existing in-market formula demonstrated to support adequate growth in infants, which is in accordance with AAP guidance [22]. We did not enroll an additional breastfed reference group for comparison. However, growth data were plotted using the WHO reference standards, which are representative of typical growth of breastfed infants. As such, the growth data reported herein were effectively compared against a breastfed reference standard.

Overall, acceptance and tolerance of study formulas were good. No differences in study discontinuation due to study formula were detected. Aside from fussiness at day 30, no significant group differences were detected in fussiness or gassiness. Mean stool frequency was not significantly different among groups by Day 120 but differed at other time points, with EHF and Control groups generally reporting more frequent stooling. Similarly, more infants in the EHF and Control groups reported “unformed or seedy” stool consistency and fewer “hard” or “formed” stools compared to infants in the AAF group.

## Conclusions

In summary, the results of the current study suggest that an EH casein formula and an AA-based formula, both with reduced protein content (2.4 g protein equivalent/100 kcal), were well tolerated and associated with normal growth in healthy, term infants from 14 to 120 days of age.

## Data Availability

The authors and study sponsor encourage and support the responsible and ethical sharing of data from clinical trials. De-identified participant data from the final research dataset used in the published manuscript may only be shared under the terms of a Data Use Agreement. Requests may be directed to the corresponding author. This study adheres to CONSORT guidelines.

## Abbreviations

AA: amino acid
AAP: American Academy of Pediatrics
CMA: cow’s milk allergy
EH: extensively hydrolyzed
